# A case report of aneurysmal subarachnoid hemorrhage in a Young with IgA: A mere co-existence?

**DOI:** 10.1016/j.ijscr.2022.106815

**Published:** 2022-02-08

**Authors:** Gagan Adhikari, Gopal Sedain, Binod Rajbhandari, Nishant Bhurtyal

**Affiliations:** a Department of General Surgery, Tribhuvan University Teaching Hospital, Institute of Medicine, Maharajgunj Kathmandu, Nepal; b Department of Neurosurgery, Tribhuvan University Teaching Hospital, Institute of Medicine, Maharajgunj Kathmandu, Nepal; c Department of Nephrology, Tribhuvan University Teaching Hospital, Institute of Medicine, Maharajgunj Kathmandu, Nepal

**Keywords:** Subarachnoid hemorrhage, IgA nephropathy, Polycystic kidney disease

## Abstract

**Introduction and importance:**

Aneurysmal Subarachnoid hemorrhage (SAH) can be associated with various other conditions like Polycystic kidney disease, Ehler Danlos syndrome, Co-arctation of the aorta, etc.

**Case presentation:**

Here we have reported a 21-year young lady with IgA nephropathy and aneurysmal SAH managed successfully.

**Clinical findings and investigations:**

The patient was a known case of IgA nephropathy diagnosed 18 months back from a kidney biopsy. She came to the emergency with **a** headache and vomiting for 2 weeks. Her initial headache was the worst she has had experienced and then the headache was continuous and not relieved by medications. Then she underwent a noncontrast CT scan of the head which showed Subarachnoid hemorrhage (SAH).

**Interventions and outcomes:**

Left pterional craniotomy and microsurgical clipping of left ICA aneurysm performed. The patient was discharged without deficits.

**Relevance and impact:**

Aneurysmal Subarachnoid hemorrhage(SAH) can be due to histopathological changes to the vessels following IgA nephropathy. These correlations could be studied further or else it could be only a mere coincidence.

## Highlights

• Aneurysmal Subarachnoid hemorrhage (SAH) can be associated with various other conditions like Polycystic kidney disease, Ehler Danlos syndrome, Co-arctation of the aorta, etc. [[1], [2], [3], [4], [5], [6], [7]]
• But can IgA nephropathy patients present with aneurysmal subarachnoid hemorrhage?

## 1 Introduction

IgA nephropathy, also known as Berger disease was first described by Berger and Hinglais in 1968 [1]. IgA (Immunoglobulin A) nephropathy is characterized by predominant IgA deposition in the glomerular mesangium [2], [3]. Regarding kidney ailments and intracranial aneurysm formation, the prevalence of un-ruptured intracranial aneurysms in the Autosomal Dominant Polycystic Kidney Disease (ADPKD) population is approximately 11% [4]. We could not find any literature regarding the association of IgA nephropathy and Intracranial aneurysms despite of search from search engines like PubMed and Google Scholar. Aneurysmal Subarachnoid hemorrhage(SAH) can be due to histopathological changes to the vessels in patients with IgA nephropathy. This case report has been reported in line with the SCARE Criteria [5].

## 2 Case

A 21-year-old lady reported to the emergency department of Tribhubhan University Teaching Hospital which is a tertiary referral center of Nepal. She presented with chief complaints of headache and vomiting for 2 weeks. Her initial headache was the worst she has had experienced and then the headache was continuous, not relieved by regular analgesics. The headache was associated with multiple episodes of vomiting. There was no history of trauma, abnormal body movement, loss of consciousness, or fever. She did not have any relevant past surgical and family history and she was not allergic to any drugs. Besides she was taking prednisolone for IgA Nephropathy. She is doesnot consume alcohol and she is nonsmoker.

She had been diagnosed with nephrotic syndrome with IgA nephropathy 18 months back for which she was taking prednisolone. Her kidney biopsy showed IgA nephropathy with global tuft sclerosis in 21.4% of glomeruli, secondary segmental sclerosis in 57.1% of capillary tufts, and a mild increase in the mesangial matrix in viable glomerular areas, patchy acute tubular injury involving viable cortical tubules, multifocal chronic interstitial inflammation and a moderate increase in tubulointerstitial chronicity. As per oxford classification of IgA nephropathy with scores of M1and S indicates mesangial hypercellularity and segmental glomerulosclerosis. There was no evidence of IgA vasculitis (Henoch Schonlein purpura). She was under regular follow-up in nephrology OPD at our center.

**Fig. 1:**
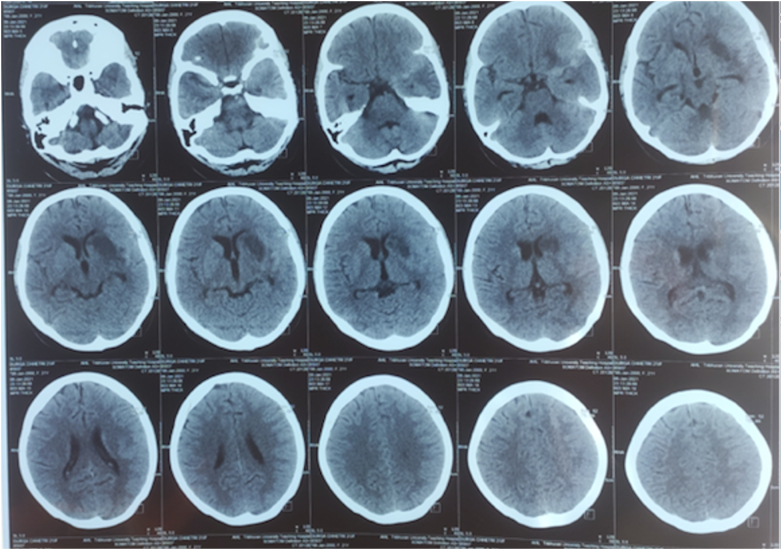
Plain CT head which shows acute subarachnoid hemorrhage involving left Sylvian fissure with hypodensity around left basal ganglia suggestive of vasospasm.

**Fig. 2:**
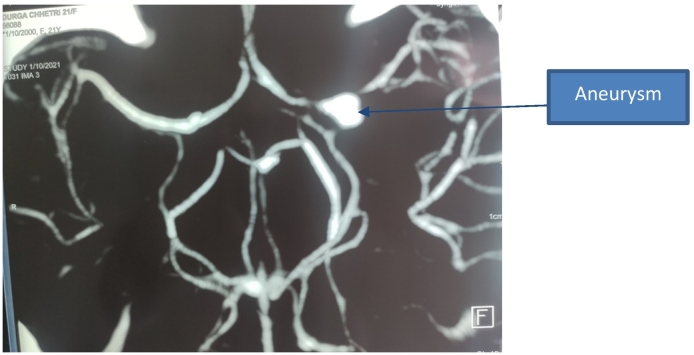
CT Angiogram shows left ICA bifurcation aneurysm measuring directed superolateral aneurysm Left pterional craniotomy and microsurgical clipping of aneurysm were performed. Intraoperative findings showed a bilobed saccular aneurysm, arising from left internal carotid artery bifurcation. The Postoperative was uneventful and the patient was discharged without deficits.

**Fig 3:**
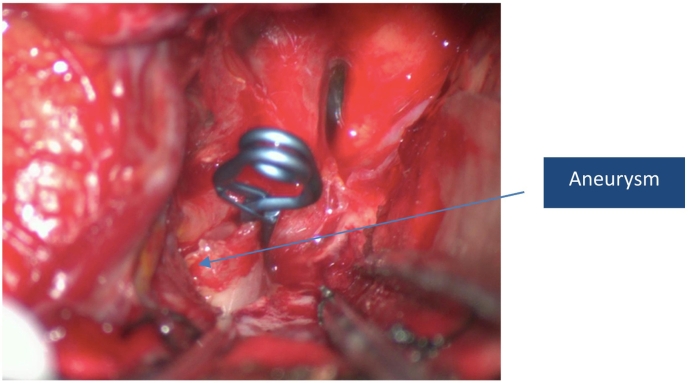
Intra op image after successful clipping of the aneurysm.

**Fig. 4:**
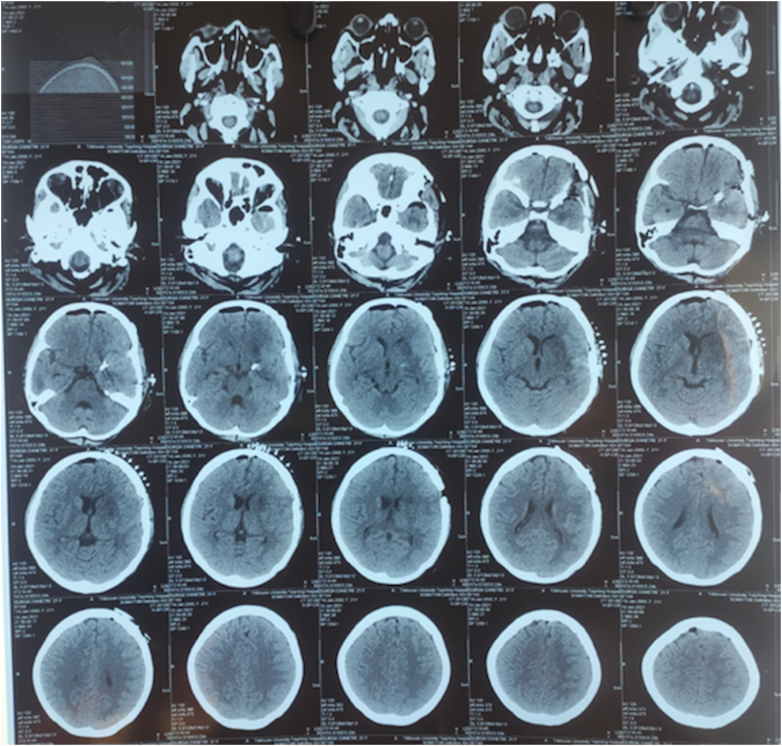
Post-op image after surgical clipping.

On examination, GCS was E4V5M6. Pupils were bilateral reactive and regular. Neck rigidity was present. She had deranged renal function during the presentation, serum urea 7.8 mmol/l and serum creatinine 216 micromol/L. Non-contrast CT head showed acute subarachnoid hemorrhage predominantly involving left Sylvian fissure. After optimization, precautions, and informed consent regarding contrast-induced nephropathy, a CT angiogram was done which revealed a left internal carotid artery bifurcation aneurysm measuring 5 × 4.9 mm with a neck measuring 2.2 mm (Fig. 1, Fig. 2, Fig 3, Fig. 4).

## 3 Discussion

In IgA nephropathy, Upstream factors (such as defective mucosal immune responses and antigen processing) directly influence one or more of the pathogenetic pathways. Specifically, the level of IgA1 bearing galactose-deficient O-glycans (Gd-IgA1) is increased in the circulation. These IgA1 glycoforms are recognized as autoantigens by antiglycan autoantibodies (anti-Gd-IgA1 autoantibodies; resulting in the formation of nephritogenic immune complexes, some of which deposit in the kidney and activate mesangial cells. Mesangial cells start to proliferate and overproduce components of the extracellular matrix, cytokines, and chemokines. Some of these cytokines can then cause podocyte injury and induce proteinuria [4]. IgA vasculitis and primary systemic vasculitis have been associated with intracranial vasculitis and aneurysm formation [6].

We hypothesize that when these immune complexes deposit in cerebral vessels, these may cause pathological changes in the vessels which may be responsible for aneurysms.

The PKD1 and PKD2 genes play an important factor in the structural integrity of vasculature hence the increased incidence of vascular diseases in ADPKD such as aneurysmal formation and spontaneous dissection [7]. There is an increased risk for the formation of aneurysms, dolichoectasia, abdominal hernias, diverticulosis, and valvular disease in these patients).

## 4 Conclusion

Intracranial aneurysms in Polycystic kidney disease is a fact. Association between IgA nephropathy and intracranial aneurysms needs to be studied further.

## Source of funding

None.

## Ethical approval

Nothing to declare.

## Consent

A written informed consent was obtained from the patient and patient's family member for publication of this case report. A copy of the written consent is available for review by the Editor-in-Chief of this journal on request.

## Guarantor

Gagan Adhikari

Department of General surgery, Tribhuvan university

Teaching Hospital, Institute of Medicine, Kathmandu, Nepal, POB: 1524.

Email: adhikarigagan2@gmail.com. Phone:+977-9851193822

## CRediT authorship contribution statement

Gagan Adhikari and Gopal Sedain: Study concept, Data collection, and surgical therapy for patient.

Gagan Adhikari: Writing and original draft preparation.

Gagan Adhikari and Gopal Sedain: Editing and writing.

Gopal Sedain, Binod Rajbhandari and Nishan Bhurtyal: senior authors and manuscript reviewer.

All the authors read and approved the final manuscript.

## Declaration of competing interest

None.
